# Multimodal MRI reveals structural and functional alterations in thyroid-associated ophthalmopathy

**DOI:** 10.3389/fneur.2026.1782025

**Published:** 2026-03-20

**Authors:** Xuejia Pu, Xiling Hu, Liyu Hu, Ke Duan, Xinbei Li, Zhujing Li, Jianzhen Lin, Yanying Wang, Jianxiang Chen, Hanqing Lyu

**Affiliations:** 1 Department of Radiology, Shenzhen Traditional Chinese Medical Hospital, Shenzhen, Guangdong, China; 2 Fourth Clinical Medical College Affiliated to Guangzhou University of Traditional Chinese Medicine, Shenzhen, Guangdong, China; 3 Department of Rehabilitation Medicine, Shenzhen People’s Hospital, Shenzhen, Guangdong, China

**Keywords:** amplitude of low-frequency fluctuation, diffusion kurtosis imaging, functional connectivity, thyroid-associated ophthalmopathy, visual pathway injury

## Abstract

**Objective:**

This study aimed to investigate white matter microstructural damage, spontaneous brain activity, and functional connectivity alterations in patients with thyroid-associated ophthalmopathy (TAO) and visual impairment using multimodal MRI.

**Methods:**

Forty-five TAO patients with visual impairment and 32 healthy controls (HCs) underwent diffusion kurtosis imaging (DKI) and resting-state functional MRI (rs-fMRI). Microstructural changes along the visual pathway were quantified using fractional anisotropy (FA), mean kurtosis (MK), and mean diffusivity (MD). Regional spontaneous activity was assessed using the amplitude of low-frequency fluctuation (ALFF) and fractional ALFF (fALFF), and seed-based functional connectivity (FC) analyses were employed. Group differences were examinded using two-sample t-tests with false discovery rate (FDR) correction (*p* < 0.05). Correlations between imaging parameters and clinical indicators were further analyzed.

**Results:**

Compared with HCs, the TAO group showed significantly decreased MK, FA, and MD values in the optic radiation, lateral geniculate body, optic tract, and optic nerve, with MK being the most markedly reduced one. Patients also exhibited increased ALFF in the right parahippocampal gyrus and decreased ALFF in the left calcarine fissure, as well as decreased fALFF in the left calcarine fissure and postcentral gyrus. Further analyses revealed decreased FC between the left calcarine fissure and the right lingual gyrus/middle frontal gyrus/bilateral postcentral gyri, and increased FC with the left supplementary motor area/fusiform gyrus. ALFF/fALFF in visual network-related regions correlated with proptosis and extraocular muscle thickening in TAO patients.

**Conclusion:**

TAO is associated with white matter microstructural damage, aberrant spontaneous neural activity, and functional reorganization. The combination of DKI and rs-fMRI (ALFF/fALFF, FC) may provide comprehensively insights into central neuropathological mechanisms, providing a basis for early diagnosis and targeted intervention.

## 1 Introduction

Thyroid-associated ophthalmopathy (TAO) is a common orbital disease, which has been ranked as the foremost disorder affecting the orbit (1). The incidence of TAO peaks between the ages of 40 and 60, with a higher prevalence observed in women than in men (2–4). Visual pathway injury is a frequent complication of TAO. This complication often develops insidiously, with early-stage symptoms typically being subtle or even unremarkable; however, in severe cases, it can potentially lead to blindness (5). Therefore, accurate and timely diagnosis, coupled with early intervention, is crucial to reduce TAO-related morbidity.

The pathological mechanisms underlying visual impairment in TAO patients are not yet fully understood. The most widely accepted mechanism is optic nerve compression due to hypertrophy of the extraocular muscles and edema of the apical orbital tissue, leading to neural damage (6). Given that the transmission and processing of visual information engage multiple central nervous system structures,including the optic nerve, optic chiasm, and optic tract—it is plausible that TAO pathophysiology may extend to the central nervous system. Recent neuroimaging studies have showen that TAO affects not only periocular structures but may also induce structure and function alterations in the visual pathway and other brain regions (7–9).

Resting-state functional magnetic resonance imaging (rs-fMRI) captures spontaneous blood oxygen level–dependent (BOLD) signals and provides a unique, noninvasive approach to characterizing brain functional architecture. It has emerged as a valuable tool for assessing the relationship between brain alterations and various ophthalmic diseases (10–13). Among rs-fMRI metrics, the amplitude of low-frequency fluctuation (ALFF) and fractional ALFF (fALFF) can quantify the intensity of regional neuronal activity, whereas functional connectivity (FC) analysis can reveal alterations in functional synchronization across distinct brain regions. These techniques have been gradually applied in investigating the central mechanisms of TAO. For example, Liu et al. (14) demonstrated abnormal spontaneous brain activity in patients with TAO using the ALFF based on rs-fMRI. Li et al. (8) utilized resting-state functional MRI and demonstrated that abnormal brain functional connectivity contributes to impaired mood and cognition in patients with hyperthyroidism. Zhang et al. (6) demonstrated significant morphological and functional alterations in the visual cortex and certain regions of the default mode network in patients with TAO and optic neuropathy (15). Jiang et al. (9), utilizing rs-fMRI, demonstrated alterations in spontaneous neuronal activity and functional connectivity patterns in patients with TAO. Nevertheless, the current understanding of visual pathway injury and associated structural and functional brain changes in TAO remains limited, highlighting a gap in effective methods for early diagnosis and objective assessment of disease severity.

Moreover, fewer studies have investigated white matter microstructural damage in the visual pathway of TAO patients. Diffusion kurtosis imaging (DKI) is an advanced diffusion MRI technique that quantifies the non-Gaussian behavior of water diffusion, enabling more sensitive detection of microstructural heterogeneity—such as axonal density and myelin integrity—in complex biological tissues like the optic nerve (16). Compared to conventional diffusion tensor imaging (DTI), DKI has demonstrated unique advantages in studies of neurodegenerative diseases and optic neuritis (17, 18). Therefore, DKI may be well suited to evaluate visual pathway damage in TAO.

Therefore, to comprehensively elucidate the neuropathological mechanisms of TAO, this study integrated DKI, ALFF/fALFF, and FC techniques to quantify microstructural integrity in various visual pathway regions, spontaneous brain activity, and visual network connectivity patterns in TAO patients and healthy controls (HCs). The study aims to: (1) quantitatively assess microstructural changes in visual pathway regions of TAO patients using DKI parameters; (2) identify key brain regions associated with abnormal cerebral activity in TAO; (3) characteriaze patterns of functional network reorganization; and (4) examine relationships between neuroimaging metrics and clinical phenotypes. The findings may provide novel insights into the central pathological mechanisms of TAO and suggest potential targets for neuromodulatory interventions.

## 2 Materials and methods

### 2.1 Participants

Patients with visual impairment due to TAO were recruited from the Departments of Ophthalmology and Endocrinology at Shenzhen Traditional Chinese Medical Hospital were enrolled. The inclusion criteria of TAO patients were: (1) Patients who meeting the diagnostic criteria for TAO (based on the Bartley criteria) (19) and presenting with visual impairment; (2) aged between 18–60 years, and (3) no contraindications to MRI.

Exclusion criteria were: (1) Individuals with visual dysfunction not attributable to TAO; (2) unable to complete the facial judgment task; (3) poorly controlled diabetes or severe systemic diseases; (4) a history of traumatic brain injury, brain tumors, or cerebrovascular disease; (5) individuals with a personal or family history of mental disorders; and (6) contraindications to the examination or MR images that did not meet quality requirements.

Finally, 45 TAO patients were included in this study (14 males and 31 females, aged 18 to 60 years, with a mean age of 38.00 ± 6.27 years). In addition, 32HCs were recruited (11 males and 21 females, aged 18 to 60 years, with a mean age of 34.32 ± 7.18 years), and were matched to the TAO group in terms of demographic characteristics such as age, gender, and educational level. Although thyroid function laboratory tests (TSH, FT3, FT4) and orbital MRI were not performed in the healthy control group, all HCs underwent detailed clinical evaluation and medical history collection to exclude: (1) history of thyroid dysfunction (including hyperthyroidism, hypothyroidism, thyroiditis, etc.); (2) history of autoimmune diseases; (3) history of orbital diseases (including proptosis, strabismus, extraocular muscle disorders, etc.); (4) history of ocular surgery or trauma. Furthermore, all HCs received routine physical examination with clinical confirmation of absence of proptosis, extraocular movement disorders, or strabismus signs.

This case–control study was approved by the Medical Ethics Committee of Shenzhen Hospital of Traditional Chinese Medicine (No. S2024-091), and written informed consent was obtained from all participants.

### 2.2 Imaging data

MRI scanning was performed on a 3.0 T Siemens Prisma scanner with a 64-channel head coil. Soft pads were placed on both sides of the participant’s neck to Results section stabilize the head and minimize motion, and earplugs were inserted into both ears to reduce noise interference. Throughout the scanning procedure, participant s were instructed to remain still with their eyes closed and to stay awake.

The following MRI sequences were acquired in both patients and HCs, including DKI, high-resolution Three-dimensional high-resolution T1-weighted structural imaging (3D-T1WI), and Resting-state blood oxygenation level-dependent functional MRI (rs-fMRI/BOLD).

DKI: Repetition time (TR) = 2,600 ms, echo time (TE) = 62 ms, slice thickness = 3 mm, and slice gap = 0 mm. Diffusion encoding was applied along 30 directions with *b*-values of 0, 1,000, and 2000 s/mm^2^.

3D-T1WI: TR = 2,200 ms, TE = 2.45 ms, inversion time (TI) = 900 ms, matrix = 256 × 256, voxel size = 1.0 × 1.0 × 1.0 mm^3^, and flip angle = 8°.

rs-fMRI(BOLD): TR = 2000 ms, TE = 30 ms, matrix = 240 × 240, 250 time points, slice thickness = 4 mm, slice gap = 0 mm, number of excitations (NEX) = 1, and flip angle = 90°.

### 2.3 Data processing and analysis

#### 2.3.1 DKI data processing and parameter quantification

DKI data were processed using the Syngo.via (https://www.siemens-healthineers.com/digital-health-solutions/syngovia) post-processing workstation. The original DICOM data were first converted to the NIfTI format and then imported into the MRIcron (https://www.nitrc.org/projects/mricron) to generate parametric maps of fractional anisotropy (FA), mean kurtosis (MK), and mean diffusion (MD). Regions of interest (ROIs) were manually delineated on the FA maps along the visual pathway. The assessed structures included the optic radiation, lateral geniculate body, optic tract, and optic nerve. The optic chiasm was excluded from evaluation due to its complex anatomy and the inherent limitations of DKI in clearly delineating this structure. These ROIs were then copied and mirrored to the contralateral side to obtain bilateral FA values. The same ROIs were used to extract MD and MK values in the corresponding regions of the visual pathway. Finally, FA, MK, and MD values were obtained for each structure (optic nerve, optic tract, lateral geniculate body, and optic radiation) and compared between the TAO group and HCs.

#### 2.3.2 fMRI data processing and analysis

The rs-fMRI data were preprocessed using the Data Processing & Analysis for Brain Imaging (DPABI, https://rfmri.org/DPABI). The steps were as follows: (1) removal of the first 10 volumes; (2) slice timing correction; (3) realignment (subjects with head motion exceeding 2 mm in translation or 2° in rotation were excluded. No subjects were excluded in this study due to excessive head motion.); (4) spatial normalization using the Diffeomorphic Anatomical Registration Through Exponentiated Lie Algebra (DARTEL) tool; (5) resampling to 3 mm isotropic voxels; and (6) spatial smoothing with a 6 mm full-width at half-maximum (FWHM) Gaussian kernel; (7) Nuisance regression was performed to remove linear and quadratic drift signals, 24 head motion parameters, and signals from white matter and cerebrospinal fluid. For resting-state functional connectivity analysis, three additional steps were applied: (1) global signal regression; (2) temporal band-pass filtering (0.01–0.1 Hz); and (3) scrubbing of volumes with framewise displacement > 0.5, along with the two preceding and one succeeding time point.

Following preprocessing, the ALFF was computed. The preprocessed BOLD time series were transformed into the frequency domain using a Fast Fourier Transform. The square root of the power spectrum was calculated and averaged across the low-frequency range (0.01–0.1 Hz) for each voxel. This ALFF value reflects the strength of regional spontaneous brain activity. fALFF was also calculated as the ratio of the power in the low-frequency range (0.01–0.1 Hz) to that of the entire frequency range (0–0.25 Hz), which helps reduce the influence of high-frequency noise and provides a more specific measure of local brain activity.

Based on the ALFF and fALFF results, the left calcarine sulcus, which showed abnormal activity, was selected as a seed region. The mean time series was extracted from this seed region. Seed-based FC analysis was then performed by computing Pearson’s correlation coefficients between the seed region’s time series and the time series of every other voxel in the brain. The resulting correlation coefficients were transformed to z-values using Fisher’s r-to-z transformation to improve normality. Finally, two-sample t-tests were conducted to compare the FC strength between the TAO patient group and the HCs.

### 2.4 Statistical analysis

Statistical analysis of demographic characteristics and clinical data was performed using SPSS software (version 22.0). To compare differences between the TAO group and HCs, independent samples t-tests were used for continuous variables with normal distribution, and chi-square tests were applied for categorical variables. For group comparisons of DKI parameters (FA, MK, MD), independent samples t-tests were used with False Discovery Rate (FDR) correction for multiple comparisons. Specifically, correction was applied separately for each parameter in each visual pathway region (optic nerve, optic tract, lateral geniculate body, optic radiation), with significance threshold set at *p* < 0.05. All reported *p*-values are FDR-corrected. For whole-brain voxel-wise ALFF and fALFF analyses, Gaussian Random Field (GRF) theory was used for multiple comparisons correction, with voxel-level *p* < 0.01 and cluster-level *p* < 0.05. This is standard practice in neuroimaging research. It should be specifically noted that the DPABI data processing software only outputs significant results passing the correction threshold (*p* < 0.05) without displaying exact p-values, which is a limitation of this software’s standard output format. Multiple comparisons correction: For ALFF and fALFF analyses, multiple comparisons were corrected using the Gaussian Random Field (GRF) theory with a voxel-level *p* < 0.01 and cluster-level *p* < 0.05. For functional connectivity analysis, the False Discovery Rate (FDR) correction was applied with a threshold of *p* < 0.05. An independent two samples t-tests were conducted to compare the differences in amplitude of ALFF, fALFF values, and FC between the TAO group and HCs with respect to clinical disease characteristics. A threshold of *p* < 0.05 was considered statistically significant.

## 3 Results

### 3.1 Comparison of demographic and clinical characteristics

No statistically significant differences were observed in gender, age, or years of education between the TAO group and the HCs (all *p* > 0.05). Best-corrected visual acuity was significantly lower in the TAO as compared to HCs (*p* < 0.05) (Table 1). The TAO group showed greater bilateral exophthalmos and thicker extraocular muscles than HCs (all *p* < 0.05) (Table 2).

**Table 1:** Comparison of general clinical characteristics.

Parameters	TAO group (*n* = 45)	Healthy controls (*n* = 32)	t/χ^2^ value	*p*
Age (years)^a^	37.00 ± 7.14	33.55 ± 7.08	1.23	0.226
Gender (Male/Female)^b^	12/20	14/8	0.98	0.252
Years of education (years)^a^	14.27 ± 4.56	15.65 ± 4.43	0.59	0.561
Best corrected visual acuity^a^	0.73 ± 0.16	0.98 ± 0.05	−8.28	**0.011** ^*^
Free triiodothyronine (FT3, pmol/L)	6.06 ± 3.2	/	/	/
Free thyroxine (FT4, pmol/L)	14.89 ± 8.27	/	/	/
Total triiodothyronine (TT3, pmol/L)	2.64 ± 2.8	/	/	/
Total thyroxine (TT4, pmol/L)	128.88 ± 24.16	/	/	/
Thyroid-stimulating hormone (TSH, mIU/L)	3.18 ± 4.46	/	/	/
TSH receptor antibody (TRAb, U/L)	13.25 ± 8.47	/	/	/

^a^For two sample *t* tests, ^b^ for X^2^, and ^*^p < 0.05 was considered statistically significant. “/” indicates data not obtained or not applicable. Thyroid function tests (FT3, FT4, TT3, TT4, TSH, TRAb) were not performed in healthy controls as they were not clinically indicated for this group. Bold values indicate *p* < 0.05, statistically significant.

**Table 2:** Comparison of exophthalmos and extraocular muscle thickness.

Parameters	TAO group (*n* = 45)	Healthy controls(n = 32)	*t* value	*p*
Exophthalmos (mm)				
Right exophthalmos	20.24 ± 1.78	15.64 ± 1.56	8.95	**<0.001** ^*^
Left exophthalmos	20.47 ± 1.83	15.33 ± 1.53	9.23	**<0.001** ^*^
Extraocular muscle thickness (mm)				
Right lateral rectus	5.72 ± 3.27	3.19 ± 0.24	3.12	**0.005** ^*^
Left lateral rectus	6.11 ± 3.15	3.12 ± 0.54	4.24	**<0.001** ^*^
Right medial rectus	4.67 ± 2.31	2.89 ± 0.56	3.42	**0.008** ^*^
Left medial rectus	4.54 ± 2.25	2.95 ± 0.58	3.31	**0.009** ^*^
Right superior rectus	4.76 ± 3.12	3.42 ± 0.63	2.83	**0.005** ^*^
Left superior rectus	4.89 ± 3.24	3.23 ± 0.69	2.69	**0.007** ^*^
Right inferior rectus	6.78 ± 4.67	3.58 ± 0.74	4.56	**<0.001** ^*^
Left inferior rectus	6.54 ± 4.21	3.55 ± 0.87	4.32	**<0.001** ^*^

Two sample *t*-tests, and ^*^p < 0.05 was considered statistically significant. Bold values indicate *p* < 0.05, statistically significant.

### 3.2 Results of DKI analysis

**Figure 1:**
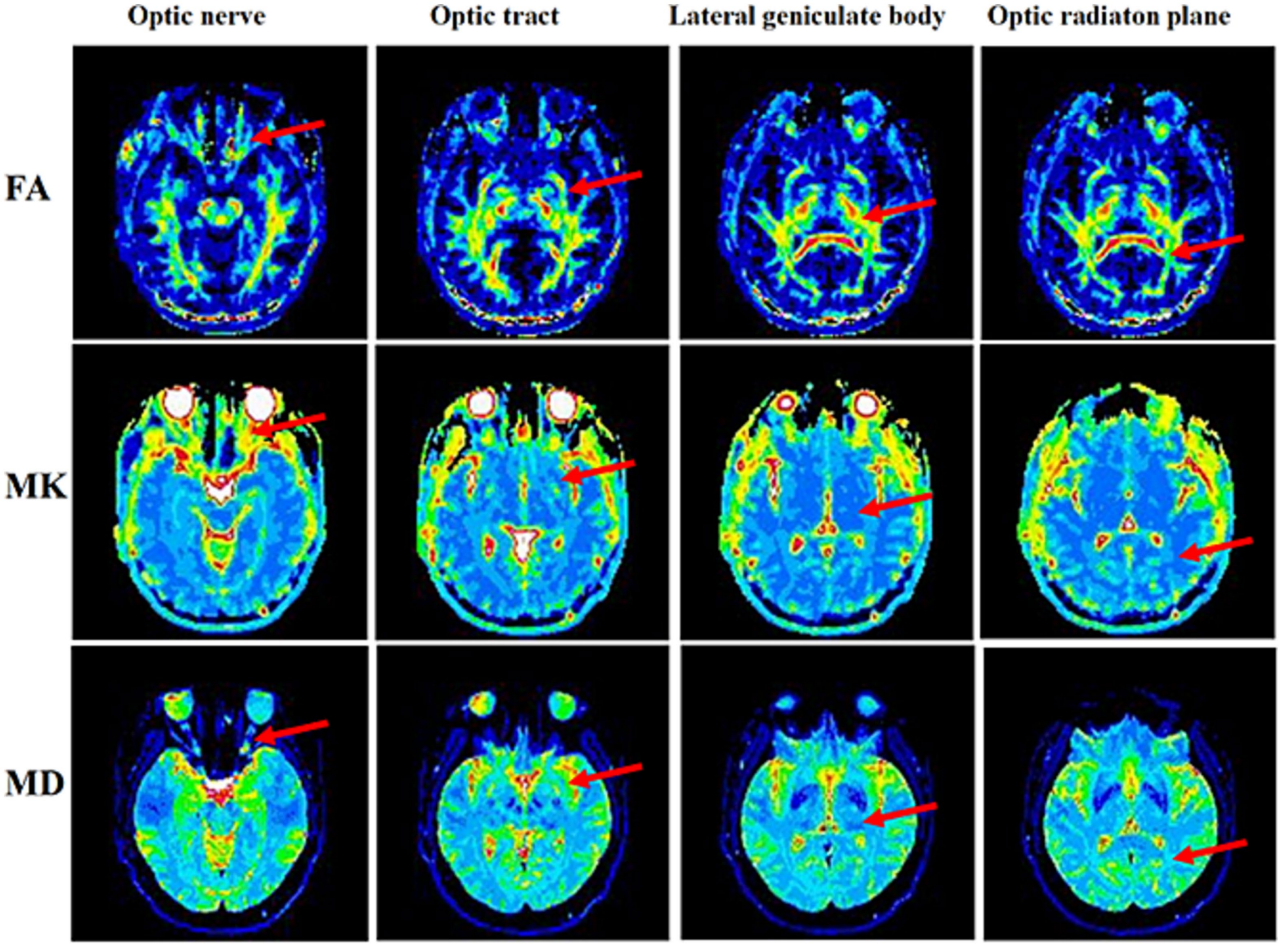
The FA, MK, and MD maps of the optic nerve, optic tract, lateral geniculate body, and optic radiation planes. FA, fractional anisotropy; MK, mean kurtosis; and MD, mean diffusion.

**Figure 2:**
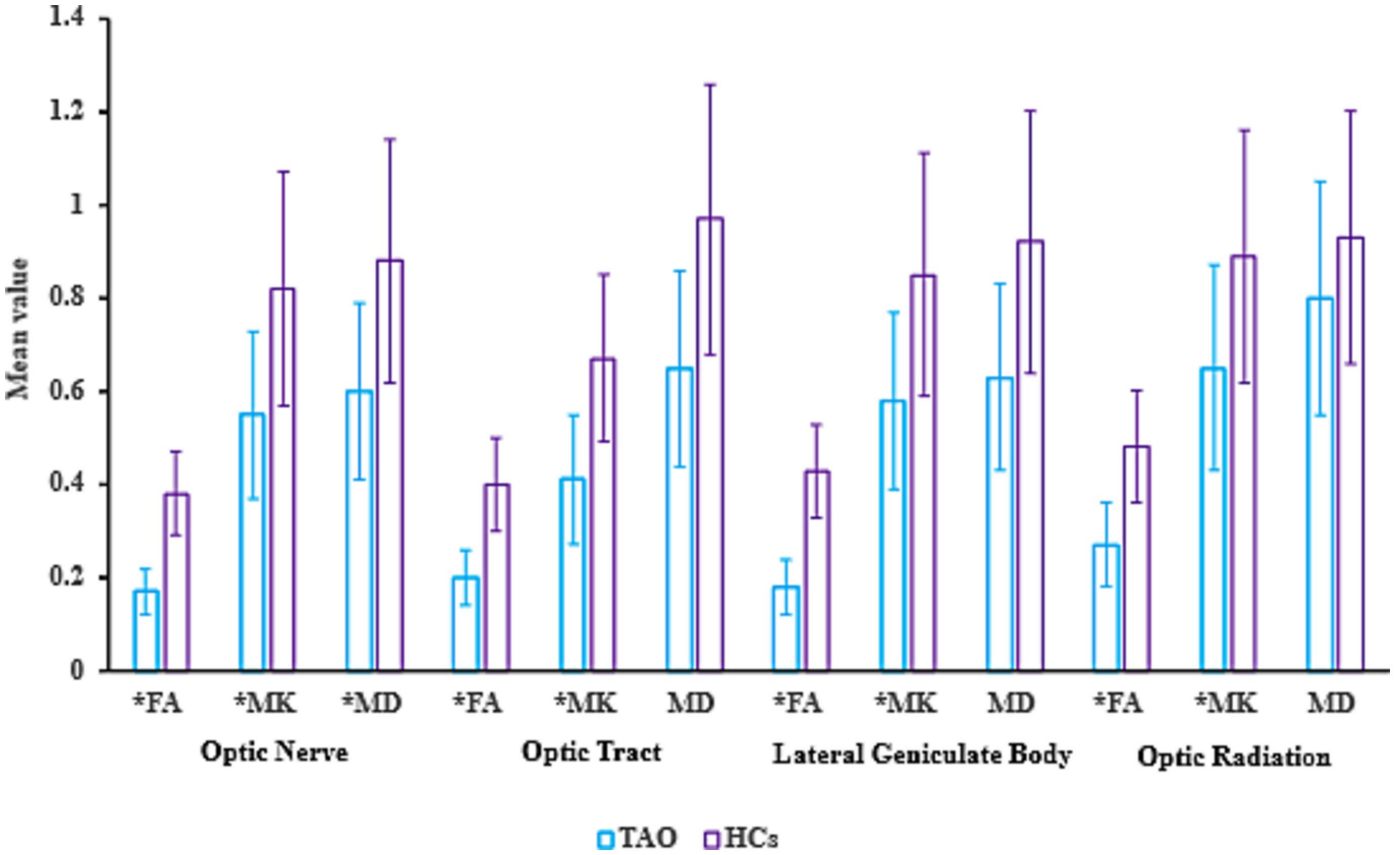
Altered DKI measurements in the optic nerve, optic tract, lateral geniculate body, and optic radiation in the TAO as compared to HCs. FA, fractional anisotropy; MK, mean kurtosis, and MD, mean diffusion; *indicates *p* < 0.05.

Measurement and analysis of the MK, FA, and MD revealed varying degrees of reduction in these metrics across seveal regions of the visual pathway (including the optic radiation, lateral geniculate body, optic tract, and optic nerve) in the TAO group compared with HCs (Figure 1). Among all the metrics, MK showed the most pronounced decrease. These findings indicate the presence of impairments of varying severity in the visual pathways of the patients (Figure 2 and Table 3).

**Table 3:** Results of DKI measurements in the optic nerve, optic tract, lateral geniculate body, and optic radiation.

Anatomical region	Parameters	TAO patients (n = 45)	Healthy controls (*n* = 32)	*p* (FDR corrected)
Optic nerve	FA value (×10^−3^ mm^2^/s)	0.17 ± 0.05	0.38 ± 0.09	**0.021** ^*^
	MK value	0.55 ± 0.18	0.82 ± 0.25	**0.008** ^*^
	MD value	0.60 ± 0.19	0.88 ± 0.26	**0.042** ^*^
Optic tract	FA value (×10^−3^ mm^2^/s)	0.20 ± 0.06	0.40 ± 0.10	**0.005** ^*^
	MK value	0.41 ± 0.14	0.67 ± 0.18	**0.003** ^*^
	MD value	0.65 ± 0.21	0.97 ± 0.29	0.543
Lateral geniculate body	FA value (×10^−3^ mm^2^/s)	0.18 ± 0.06	0.43 ± 0.10	**0.012** ^*^
	MK value	0.58 ± 0.19	0.85 ± 0.26	**0.005** ^*^
	MD value	0.63 ± 0.20	0.92 ± 0.28	0.724
Optic radiation	FA value (×10^−3^ mm^2^/s)	0.27 ± 0.09	0.48 ± 0.12	**0.026** ^*^
	MK value	0.65 ± 0.22	0.89 ± 0.27	**0.007** ^*^
	MD value	0.80 ± 0.25	0.93 ± 0.27	0.745

Two sample *t*-tests, and *p < 0.05 was considered statistically significant. Bold values indicate *p* < 0.05, statistically significant.

### 3.3 Results of the ALFF analysis

**Figure 3:**
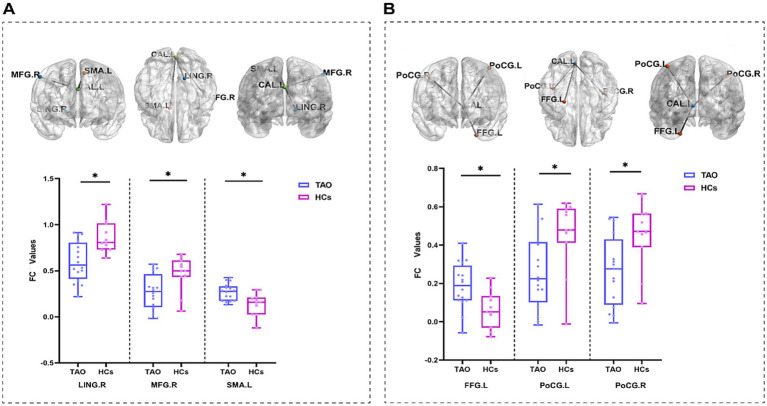
**(A)** Brain regions with ALFF differences between TAO patients and HCs; **(B)** Brain regions with fALFF differences between TAO patients and HCs. CAL. L, left calcarine sulus; PHG. R, right parahippocampal gyrus; and PoCG. L, left postcentral gyrus; *indicates *p* < 0.05.

In the statistical analysis, with age, gender, and framewise displacement (a head motion parameter) included as covariates, comparisons were conducted between the TAO group and HCs. Multiple comparisons were corrected using the GRF (voxel-level *p* < 0.01, cluster-level *p* < 0.05). Compared with HCs, the TAO group exhibited a significant increase in ALFF in the right parahippocampal gyrus and a significant decrease in the ALFF in the left calcarine sulcus (Figure 3A and Table 4). In addition, the fractional ALFF (fALFF) was significantly decreased in the left calcarine sulcus and the left postcentral gyrus (Figure 3B and Table 4).

**Table 4:** Brain regions with ALFF/fALFF differences between TAO patients and HCs.

Metric	Cluster	Brain region	MNI coordinates (x, y, z)	Cluster size (voxels)	*T*
ALFF	Cluster 1	Right parahippocampal gyrus	(33,-39,-12)	242	5.2684
	Cluster 2	Left calcarine sulcus	(0,-87,3)	139	−4.1924
fALFF	Cluster 1	Left calcarine sulcus	(−6,-81,3)	109	−4.0680
	Cluster 2	Left postcentral gyrus	(−51,-21,45)	109	−3.8297

Montreal Neurological Institute (MNI) space; coordinates (X, Y, Z) of the peak activation in MNI space; peak (*T*-value).

### 3.4 Results of functional connectivity analysis

**Figure 4:**
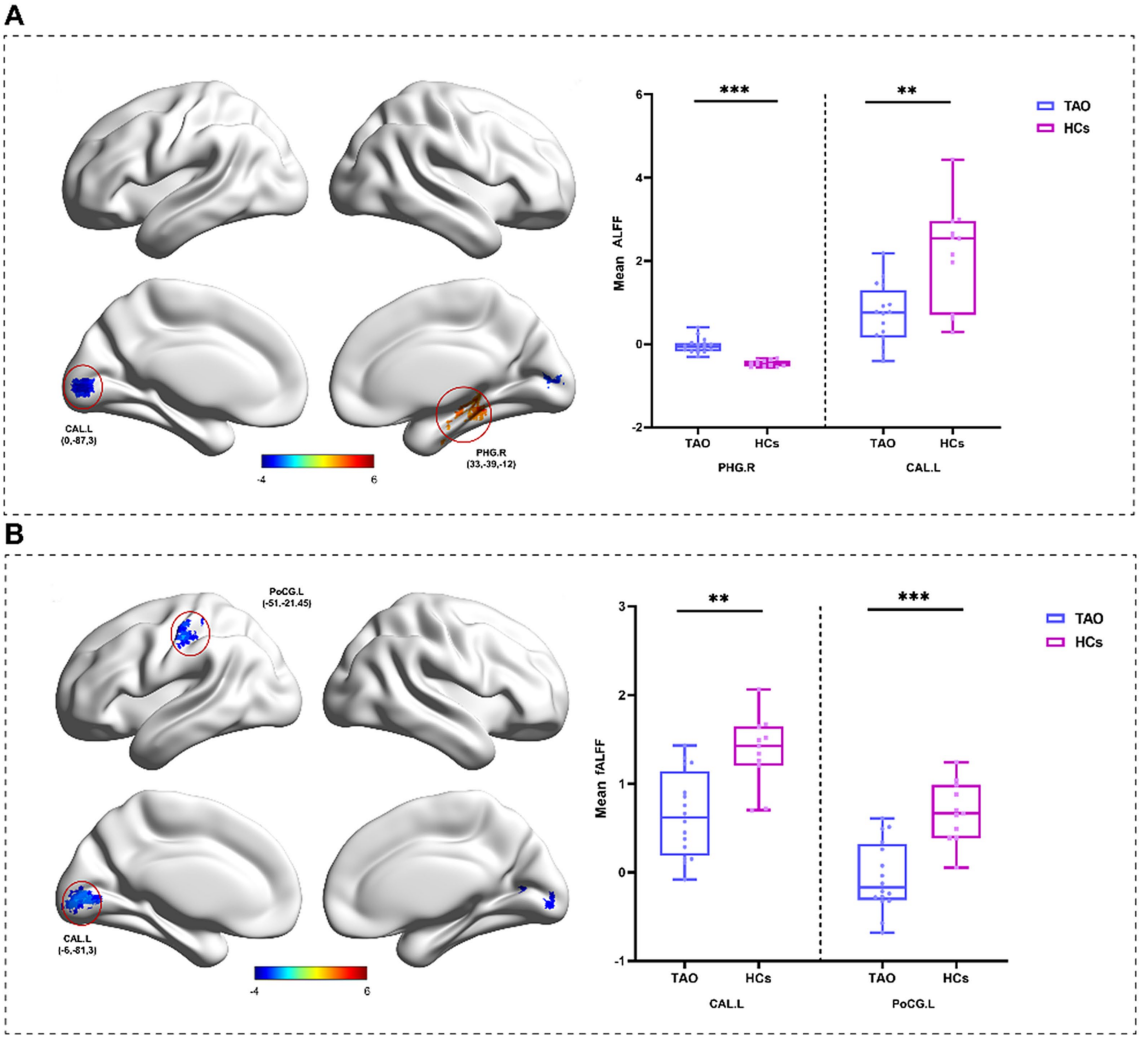
Comparison between patients with TAO and healthy controls (HCs). **(A)** Represents the brain regions showing differences in whole-brain functional connectivity using the calcarine sulcus as a seed region based on the ALFF metric. **(B)** Represents the brain regions showing differences in whole-brain functional connectivity using the calcarine sulcus as a seed region based on the fALFF metric. L (left); R (right); CAL, calcarine sulcus; FFG, fusiform gyrus; LING, lingual gyrus; MFG, middle frontal gyrus; PoCG, postcentral gyrus; and SMA, supplementary motor area; *indicates *p* < 0.05; **indicates *p* < 0.01; ***indicates *p* < 0.001.

In the FC analysis, the false discovery rate (FDR) correction was applied for multiple comparisons, with the statistical significance set at *p* < 0.05. Since the primary visual cortex is located in the cortical area surrounding the calcarine sulcus, the two previously identified calcarine sulcus regions showing significant differences were used as seed masks for further seed-based FC analysis. This analysis aimed to compare differences in functional connectivity between these visual brain regions (which exhibited alterations in spontaneous brain activity in patients) and other brain regions. Compared to HCs, the TAO group exhibited increased FC between the left calcarine sulcus and the left supplementary motor area (SMA) and the left fusiform gyrus, as well as decreased FC between the left calcarine sulcus and the right lingual gyrus, right middle frontal gyrus, and bilateral postcentral gyri (Figure 4 and Table 5).

**Table 5:** Detailed results of seed-based functional connectivity analysis.

Seed Region	Connected brain region	MNI coordinates (x, y, z)	Cluster size (voxels)	Peak *T*	*p* (FDR corrected)
Left calcarine sulcus (ALFF)	Right lingual gyrus	(−24, −12, −39)	223	4.6495	**0.002**
	Right middle frontal gyrus	(−42, −36, 63)	273	−4.4005	**0.003**
	Left supplementary motor area	(42, −27, 48)	354	−4.4611	**0.002**
Left calcarine sulcus (fALFF)	Left fusiform gyrus	(15, −48, −3)	252	−4.742	**< 0.001**
	Left postcentral gyrus	(54, −9, 51)	421	−4.3607	**0.001**
	Right postcentral gyrus	(−9, 6, 57)	238	4.5509	**0.002**

Positive *T*-values indicate increased FC in TAO patients compared to HCs; negative *T*-values indicate decreased FC; Seed region: left calcarine sulcus selected based on ALFF/fALFF analysis results (regions showing abnormal spontaneous activity); FDR-corrected *p*-values were estimated based on peak *T*-values and degrees of freedom (df = 75) using the Benjamini-Hochberg procedure. DPABI software does not output exact *p*-values for FC analysis; these values represent conservative estimates derived from *post-hoc* calculations. All reported connections survived FDR correction at *p* < 0.05; *p < 0.05 was considered statistically significant. Bold values indicate *p* < 0.05, statistically significant.

### 3.5 Correlation between brain regions with altered FC and clinical disease characteristics

In the TAO group, significant correlations were identified only between FC and exophthalmos degree/extraocular muscle thickening, whereas no significant correlations were observed between ALFF/fALFF/DKI measurements and exophthalmos degree or extraocular muscle thickening (Table 6).

**Table 6:** Results of the correlation analysis between altered FC and clinical disease characteristics in the TAO.

Seed point (Mask)	Brain regions with differences	Clinical features	Correlation coefficient	*p* (FDR corrected)
(Left calcarine sulcus) Based on ALFF metric	Right lingual gyrus	Degree of exophthalmos (Left)	0.590	**0.002** ^*^
		Degree of exophthalmos (Right)	0.659	**<0.001** ^*^
		Left lateral rectus muscle	0.490	**0.014** ^*^
		Right inferior rectus muscle	0.448	**0.028** ^*^
	Right middle frontal gyrus	Degree of exophthalmos (Left)	0.446	**0.029** ^*^
		Degree of exophthalmos (Right)	0.500	**0.013** ^*^
	Left supplementary motor area	Degree of exophthalmos (Left)	0.726	**<0.001** ^*^
		Degree of exophthalmos (Right)	0.731	**<0.001** ^*^
		Left lateral rectus muscle	0.480	**0.017** ^*^
		Right inferior rectus muscle	0.579	**0.003** ^*^
(Left calcarine sulcus) Based on fALFF metric	Left fusiform gyrus	Degree of exophthalmos (Left)	0.642	**0.001** ^*^
		Degree of exophthalmos (Right)	0.675	**<0.001** ^*^
		Left lateral rectus muscle	0.456	**0.025** ^*^
		Right inferior rectus muscle	0.491	**0.015** ^*^
	Left postcentral gyrus	Degree of exophthalmos (Left)	0.442	**0.031** ^*^
		Degree of exophthalmos (Right)	0.501	**0.013** ^*^
	Right postcentral gyrus	Degree of exophthalmos (Left)	0.484	**0.016** ^*^
		Degree of exophthalmos (Right)	0.534	**0.007** ^*^

FDR, false discovery rate. *p < 0.05 was considered statistically significant. Bold values indicate *p* < 0.05, statistically significant.

## 4 Discussion

Using DKI and rs-fMRI, this study revealed patterns of white matter microstructural damage in the visual pathway, aberrant regional brain activity, and abnormal visual network connectivity in patients with TAO. Compared with HCs, the TAO group showed varying degrees of reduction in FA, MK, and MD in specific regions of the visual pathway, including the optic nerve, optic tract, lateral geniculate body, and optic radiation, with the decrease in MK values being the most pronounced one. Furthermore, the TAO group showed decreased ALFF and fALFF in key regions such as the left calcarine sulcus and left postcentral gyrus, as well as an increased ALFF in the right parahippocampal gyrus. FC analysis further indicated reduced connectivity between the left calcarine sulcus and nodes of the visual and sensorimotor networks (e.g., right lingual gyrus, bilateral postcentral gyri, right middle frontal gyrus), but increased connectivity with SMA and the left fusiform gyrus in TAO patients.

This study showed that FA, MK, and MD values were decreased to varying degrees in specific regions of the visual pathway, including the optic nerve, optic tract, lateral geniculate body, and optic radiation, in the TAO group, with the most pronounced reduction observed in MK. The MK value reflects the heterogeneity and complexity of the tissue microstructure. Its decrease suggests impaired axonal membrane integrity and reduced complexity of the micro-environment, potentially associated with mechanical compression of the optic nerve or axonal degeneration resulting from intraorbital inflammatory responses in TAO patients. Previous studies have indicated that hyperplasia of intraorbital fat and enlargement of extraocular muscles in TAO patients can elevate pressure within the optic nerve sheath, leading to impaired axonal transport and mitochondrial dysfunction, ultimately causing axonal atrophy (5, 12, 19). The decrease in FA may be related to myelin loss or extracellular edema; however, this interpretation requires validation with histopathological evidence. These findings are consistent with the pathological mechanisms of dysthyroid optic neuropathy (DON), indicating that visual pathway damage in TAO patients is not confined to the intraorbital segment of the optic nerve but may extend centrally through the optic chiasm and optic radiations, forming a multi-segment injury pattern. Future research should involve longitudinal tracking to observe the dynamic evolution of the visual pathway’s microstructure, clarifying the reversibility of the damage and identifying optimal intervention windows.

The occipital lobe contains the brain’s primary visual areas, and the calcarine sulcus is the most reliable anatomical landmark in the medial occipital lobe (20). A prior fALFF study comparing patients with TAO and HCs revealed reduced spontaneous neuronal activity in the right calcarine sulcus region of TAO patients during the active phase of the disease (21). Another fALFF study comparing patients with TAO and HCs revealed reduced activity in the bilateral calcarine sulcus regions (22). Our findings demonstrated that TAO patients exhibited significantly decreased ALFF/fALFF values in the left calcarine sulcus (primary visual cortex), potentially arising from long-term abnormal visual input (such as diplopia caused by proptosis and restricted extraocular muscle movement). These results suggest decreased spontaneous neuronal activity in visual cortical regions, indicating impaired visual information processing. Research indicates that chronic and inefficient signal input from the visual pathway can lead to an adaptive downregulation of neural activity in the primary visual cortex, as a mechanism to reduce energy consumption (23). In this study, the weakened FC between the left calcarine sulcus and the right lingual gyrus (a visual association cortex) may further impair the integration of visual information. This phenomenon is similar to the visual network segregation pattern observed in patients with glaucoma (24).

The postcentral gyrus is part of the parietal lobe, housing the primary somatosensory cortex, which comprises Brodmann areas 1, 2, and 3 (25). An fMRI study on TAO reported a significant decrease in ALFF in the left postcentral gyrus, along with reduced FC within this region (9). Our results indicated that the decreased fALFF in the left postcentral gyrus (primary somatosensory cortex) may be associated with TAO-related chronic periorbital pain or facial paresthesia. The parahippocampal gyrus is involved in memory, cognition, emotion, and visualspatial processing (26, 27). The increased fALFF in the right parahippocampal gyrus may reflect a compensatory adjustment for emotional and cognitive functions.

The weakened FC between the left calcarine sulcus and the right middle frontal gyrus (which participates in visual attention regulation) may impair top-down attentional allocation, leading to reduced processing efficiency for complex visual scenes in patients. In contrast, the enhanced FC between the left calcarine sulcus and the left fusiform gyrus (a key region for face and object recognition) could be attributed to two potential mechanisms: (1) a compensatory adaptation to optimize visual information processing, possibly in response to impaired visual input (such as diplopia, visual field deficits, or blurred vision) in TAO patients, prompting the primary visual cortex (calcarine sulcus) to strengthen its link with higher-order visual processing areas (fusiform gyrus); and/or (2) systemic inflammation in TAO may affect the central nervous system by crossing the blood–brain barrier, potentially promoting the expression of synaptic plasticity–related molecules and thereby strengthening functional connections.

The enhanced FC between the left calcarine sulcus and the left supplementary motor area (involved in motor planning and coordination) may correspond to an adaptive motor strategy developed by TAO patients to compensate for restricted eye movement. For example, patients might rely on increased head–neck coordination to offset the reduced range of extraocular muscle activity, a behavioral adaptation requiring cross-modal collaboration between the supplementary motor area and the visual cortex (28).

Our study found that the ALFF/fALFF in visual network-related brain regions (such as the calcarine sulcus and postcentral gyrus) were significantly correlated with the degree of exophthalmos and extraocular muscle enlargement, suggesting that these neuroimaging metrics may serve as biomarkers for assessing TAO severity. A multimodal integrated analysis combining visual pathway microstructure parameters (e.g., MK values) and functional network indicators could help develop predictive models for neurological damage in TAO, thereby providing an evidence basis for personalized treatment decisions (such as immunosuppressive therapy or optic nerve decompression surgery).

This study has several limitations. The relatively small sample size may have reduced the statistical power. The potential influence of fluctuations in thyroid hormone levels on brain activity was not controlled for. Furthermore, molecular imaging or pathological evidence was not available to substantiate the findings. As an observational case–control study, the design precludes definitive conclusions regarding causal relationships between the visual pathway damage/functional brain alterations and the disease course. Future research should involve longitudinal follow-up studies, incorporate measurements of serum inflammatory factors, and utilize animal models to validate the underlying pathological mechanisms. Expanding the sample size and performing subgroup analyses stratified by disease stage are necessary to clarify the central pathological features at different phases of the illness. Additionally, integrating multi-omics technologies with machine learning could help construct a multidimensional “structure–function–metabolism” predictive framework for TAO. One limitation of this study is that thyroid function laboratory tests and orbital imaging were not performed in the healthy control group. Although we excluded obvious thyroid and orbital diseases through detailed history collection and clinical examination, we cannot completely rule out the possibility of subclinical thyroid dysfunction. Future studies should include laboratory screening of controls to improve the specificity of between-group comparisons.

In summary, patients with TAO exhibit not only microstructural damage to the white matter in the visual pathway but also abnormal spontaneous neural activity in multiple brain regions and functional network reorganization. DKI can sensitively detect early microstructural alterations within the visual pathway. Combining DKI with multimodal rs-fMRI indices, such as ALFF/fALFF and FC, may provide complementary insights into the central neuropathological mechanisms of TAO. This integrated approach provides a theoretical foundation for early diagnosis and targeted intervention strategies.

## Data Availability

The raw data supporting the conclusions of this article will be made available by the authors, without undue reservation.
